# OP08 A Comprehensive Collaborative Framework To Enhance Orphan Drug Accessibility For Rare Diseases: Insights From Türkiye

**DOI:** 10.1017/S0266462325100676

**Published:** 2025-12-29

**Authors:** Birol Tibet, Selma Karabey, Ayse Emel Onal, Ugur Ozbek

## Abstract

**Introduction:**

Restricted access to orphan drugs significantly challenges rare disease patients, impairing health outcomes and quality of life. In Türkiye, systemic barriers such as regulatory inefficiencies, economic limitations, and inadequate stakeholder collaboration persist. This study evaluated these issues through stakeholder perspectives, proposing a collaborative framework to improve access, ensure equity, and enhance systemic efficiency in addressing rare disease challenges in Türkiye.

**Methods:**

This qualitative study employed semi-structured focus group discussions with 19 patients and caregivers and 13 in-depth interviews with healthcare providers, policymakers, industry representatives, and regulatory bodies. Thematic analysis of 1,996 coded excerpts identified key barriers to orphan drug access, categorized as direct or indirect impacts. Grounded theory was applied to synthesize these findings into a cohesive framework addressing economic, regulatory, and systemic challenges. Data triangulation enhanced validity, ensuring the perspectives of diverse stakeholders were accurately represented. Stakeholder feedback on the framework was incorporated iteratively, ensuring practicality and adaptability for policy and decision-making in Türkiye. Supported by TUBITAK 2224-A Program for participation in HTAi 2025; it had no role in the study conduct.

**Results:**

The study revealed multifaceted barriers to orphan drug access in Türkiye, including fragmented regulatory processes, high costs, and inadequate inter-stakeholder collaboration. Indirect factors, such as limited public awareness and insufficient professional education, exacerbated these challenges. The proposed framework emphasizes centralized regulatory mechanisms to streamline approvals, public–private partnerships to mitigate financial constraints, and enhanced communication to ensure transparency and accountability. Stakeholder feedback highlights the framework’s adaptability and potential impact, particularly in addressing disparities within Türkiye. Success metrics, such as improved regulatory efficiency and patient outcomes, were defined to support future implementation and continuous improvement.

**Conclusions:**

The proposed framework addresses critical systemic barriers to orphan drug access in Türkiye by fostering collaboration, streamlining regulatory pathways, and enhancing equity. It emphasizes the importance of stakeholder alignment and sustainable policy solutions for rare disease patients. Future research may focus on piloting the framework, adapting it for broader contexts, and developing evaluation tools to measure its long-term impact.

